# Mutant Prevention Concentrations of Imipenem and Meropenem against *Pseudomonas aeruginosa* and *Acinetobacter baumannii*

**DOI:** 10.1155/2014/979648

**Published:** 2014-12-30

**Authors:** E. Dahdouh, S. H. Shoucair, S. E. Salem, Z. Daoud

**Affiliations:** Clinical Microbiology Laboratory, Faculty of Medicine & Medical Science, University of Balamand, P.O. Box 100, Tripoli, Lebanon

## Abstract

The aim of this study was to determine the usefulness of the MPC of carbapenems against clinical isolates of* Pseudomonas *spp. and* Acinetobacter *spp. and to assess its possible relationship with mechanisms of resistance. Detection of the mechanisms of resistance was performed using Antibiotic Susceptibility Testing, Double Disk Synergy, disk antagonism, addition of NaCl to the medium, addition of PBA or EDTA to Carbapenem disks, addition of PBA to Cefoxitin disks, and CCCP test for 10* Pseudomonas *spp. and* Acinetobacter baumannii* strains. The MIC and MPC were determined using the broth macrodilution and plate dilution methods, respectively. Four *Acinetobacter baumannii* strains produced MBL. Two of them produced Oxacillinase and one produced ESBL. Two* Pseudomonas *spp. isolates produced both KPC and MBL. The resistant* Acinetobacter *spp. and* Pseudomonas *spp. strains had higher MPC values than susceptible ones. However, the Mutant Selection Window was found to be dependent on the degree of resistance but not on a particular mechanism of resistance. The usefulness of the MPC was found to be dependent on its value. Based on our data, we recommend determining the MPC for each isolate before using it during treatment. Furthermore, the use of T>MSW instead of T>MIC is suggested.

## 1. Introduction

*Pseudomonas* spp. and* Acinetobacter *spp. are opportunistic bacteria that are being increasingly implicated with severe nosocomial infections [1, 2]. Carbapenems are commonly used for the treatment of infections with these organisms for critically ill patients [3]. However, reports of carbapenem resistance among these bacteria are increasing worldwide [4, 5].

*Pseudomonas *spp. and* Acinetobacter *spp. may show carbapenem resistance through several mechanisms. These mechanisms include changes in their outer membrane, production of efflux pumps, and production of *β*-lactamases that are able to hydrolyze carbapenems [6]. Several approaches are aimed at restricting the emergence of resistance throughout the course of treatment in order to improve treatment outcomes and safeguard antibiotics for future use [7]. One such approach is the implementation of the Mutant Prevention Concentration (MPC) [8].

The MPC is a pharmacodynamic parameter that is aimed at suppressing the emergence of resistance throughout antimicrobial treatment. It is defined as the concentration that prevents the growth of first step mutants [9]. This parameter has been shown to successfully suppress the emergence of resistance for concentration dependent antibiotics [7]. The Mutant Selection Window (MSW) is the difference between the MPC and the MIC and it describes the concentration range in which microorganisms are encouraged to mutate and are selected for [9]. In terms of its use for time dependent antibiotics, one study concludes that the MPC is of little significance for these antibiotics [10] while another study concludes that it is worthy of further investigation [11]. It is our aim to investigate the MPC parameter in light of the different mechanisms of resistance detected by phenotypic tests and to further evaluate its role in the suppression of carbapenem resistance during treatment.

## 2. Materials and Methods

A total of 5 clinical isolates of* Pseudomonas *spp. and 5* Acinetobacter *spp. were tested. The Minimum Inhibitory Concentration (MIC) was determined according to the CLSI guidelines for Imipenem and Meropenem using the broth macrodilution method [12]. The MPC was determined using the agar plate dilution method as previously described for the same antibiotics [13]. Briefly, the antimicrobial agents were incorporated into Mueller Hinton Agar (MHA) plates so as to create a concentration gradient from 1000 *μ*g/mL to 0.1 *μ*g/mL. The bacterial suspension was then concentrated to 10^10^ CFU/mL by centrifugation at 3000 g for 15 min and inoculated on the entire range of antibiotic containing MHA plates. The plates were incubated for 48 hours and checked for growth at 24 and 48 hours. The concentration of the first plate that showed no growth after 48 hours was considered as the MPC. All the tests were performed in duplicate.

The Antibiotic Susceptibility Testing (AST) for all the strains was performed according to CLSI guidelines [12]. ESBL production among the tested strains was determined using the Double Disk Synergy Test (DDST) [14]. Chromosomal and plasmidic AmpC production was determined using the disk antagonism test [15] and the increase of the inhibition zone of the Cefoxitin Disk upon PBA induction [16], respectively. KPC and MBL production were determined via the addition of PBA and EDTA, respectively, to Imipenem and Meropenem disks. An increase in the inhibition zone of these antibiotic disks of 5 mm or more upon the addition of these chemicals was considered positive for the respective enzyme [17]. OXA production was detected by determining the MIC with and without 200 mM NaCl in the testing medium. A 4-fold decrease in MIC upon NaCl addition was considered positive for OXA production [18]. Efflux pump overproduction was determined by incorporating CCCP, an inhibitor of efflux pumps, into MHA plates and detecting a change of 5 mm or more in the inhibition zone of Imipenem and Meropenem disks with and without this chemical [19].

## 3. Results

The results of the AST are shown in Tables 1 and 2.* Acinetobacter baumannii *strains A2, A3, A4, and A5 and* Pseudomonas *spp. strains P4 and P5 were resistant to carbapenems. Strain A4 was the only* Acinetobacter baumannii* strain that was susceptible to Ceftazidime, Cefepime, Gentamycin, Ciprofloxacin, Amikacin, and Trimethoprim Sulfamethoxazole. None of the* Acinetobacter baumannii *strains were susceptible to Cefotaxime, Cefuroxime, Cefoxitin, Amoxicillin + Clavulanic acid, and Ampicillin. However, they all appeared to be susceptible to Tigecycline. Among the* Pseudomonas *spp., only strains P3 and P4 were susceptible to Aztreonam. Strain P2 was resistant to Gentamycin, Tobramycin, Ceftazidime, and Ciprofloxacin and strain P5 was resistant to Ceftazidime and Ciprofloxacin. Strains P2, P3, and P5 were resistant to Piperacillin but only strain P5 was resistant to Piperacillin/Tazobactam. Moreover, all the tested strains were resistant to Trimethoprim Sulfamethoxazole but susceptible to Amikacin.

The results of the phenotypic tests are shown in Table 3.* Acinetobacter baumannii* strain A4 was positive for ESBL production. All 4 carbapenem resistant* Acinetobacter baumannii *strains (A2, A3, A4, and A5) expressed MBL and only strains A2 and A3 expressed OXA. Among the* Pseudomonas *spp., strains P3, P4, and P5 showed chromosomal AmpC production while only strain P5 showed plasmidic AmpC production. The carbapenem resistant strains P4 and P5 expressed both MBL and KPC. None of the tested strains was positive for efflux pump overproduction.

The MIC, MPC, and MSW values for Imipenem and Meropenem are shown in Tables 4 and 5, respectively. The data using Imipenem shows that* Acinetobacter baumannii *strains had a higher average MPC (255.2 *μ*g/mL) and MSW (219.6 *μ*g/mL) than* Pseudomonas *spp. (48 *μ*g/mL and 40.5 *μ*g/mL resp.). Strain A4 had the highest MSW (672 *μ*g/mL) while strain A1 showed the lowest MSW (2 *μ*g/mL). The average MSW of resistant* Acinetobacter baumannii *strains (274 *μ*g/mL) was much higher than that of susceptible strains (2 *μ*g/mL). Resistant* Pseudomonas *spp. strains also had a higher average MSW (79 *μ*g/mL) than susceptible strains (14.38 *μ*g/mL).

**Figure 1:**
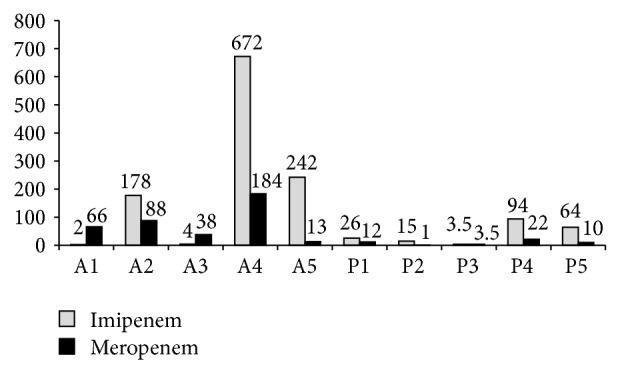
Mutant Selection Window for 5* Acinetobacter baumannii* and 5* Pseudomonas *spp. strains using Imipenem and Meropenem.

Using Meropenem,* Acinetobacter baumannii *strains showed higher average MPC (101 *μ*g/mL) and MSW (77.8 *μ*g/mL) values than* Pseudomonas *spp. (13.5 *μ*g/mL and 9.7 *μ*g/mL resp.). The highest MSW observed was for strain A4 (184 *μ*g/mL) and the smallest MSW observed was for strain P2 (1 *μ*g/mL). A higher average MSW for resistant* Acinetobacter* strains (80.75 *μ*g/mL) as compared to susceptible strains (66 *μ*g/mL) was also observed for this antibiotic. Similarly, resistant* Pseudomonas* strains had a higher MSW average (16 *μ*g/mL) than susceptible strains (5.5 *μ*g/mL). The MSW for each individual strain is shown in Figure 1.

## 4. Discussion

*Pseudomonas aeruginosa *and* Acinetobacter baumannii* are among the most commonly isolated nosocomial pathogens worldwide [20]. Carbapenems are being increasingly used for the treatment of infection with these organisms [21]. However, rates of carbapenem resistance among these pathogens are escalating all over the world [5, 22, 23]. The Pharmacodynamic/Pharmacokinetic approach is commonly used for the optimization and individualization of the treatment of critically ill patients [24]. Nevertheless, there is a rising trend to use this approach for minimizing the emergence of resistance as well [25]. The MPC was found to be a good pharmacodynamic parameter that helps in suppressing the emergence of resistance as far as concentration dependent antibiotics are concerned [7]. For time-dependent antibiotics, the importance of the MPC does not lie in providing a concentration that could increase the killing rate of the antibiotic but in defining the upper border of the MSW. The MSW is the concentration range whose upper limit is the MPC and lower limit is the MIC, and it is where resistant strains are selected for [26]. Maintaining antibiotic concentrations above the MSW throughout the course of treatment could lead to the suppression of the emergence of resistance and ultimately better treatment outcomes.

In this study, we investigated if there was a correlation between the phenotypic detection of the mechanisms of resistance and the MPC values in order to provide routine medical laboratories with a tool with which the MSW could be predicted. The mechanisms of resistance were phenotypically detected in 5 clinical isolates of* Pseudomonas *spp. and 5 clinical isolates of* Acinetobacter baumannii*. MBLs, KPCs, and OXAs are carbapenemases that are being increasingly responsible for carbapenem resistance among* Pseudomonas *spp. and* Acinetobacter baumannii* isolates [6, 27]. Several studies from Latin America, the United States of America, and China have shown an increase in KPC production among* Pseudomonas aeruginosa* isolates [28, 29]. MBL and OXA production among* Acinetobacter *spp. have been reported in Australia, the United States of America, Tahiti, China, Korea, Libya, Pakistan, Latin America, and several countries across Europe [4, 30, 31]. Among the tested strains, MBL has been detected in* Acinetobacter *spp. strains A2, A3, A4, and A5. In addition, OXA production was detected in strains A2 and A3. This finding is not uncommon since OXA and MBL have been reported to be coexpressed in isolates from Spain, Greece, Singapore, and Australia [31]. Among the* Pseudomonas *spp. isolates, strains P4 and P5 were both found to coexpress MBL and KPC. None of the tested strains were positive on the CCCP test. However, this does not exclude the possibility of having other nonenzymatic mechanisms of resistance acting in conjunction of the detected mechanisms to produce carbapenem resistance for any of the tested strains. These mechanisms may include the downregulation of outer membrane proteins and the presence of certain Penicillin Binding Protein subtypes that have a low affinity to carbapenems [32, 33]. Nevertheless, there are no phenotypic tests that could be used in routine clinical laboratory testing to detect such changes. Since this study is aimed at evaluating the MPC in light of the phenotypic tests that could be easily performed in clinical laboratories, further molecular investigation was not undertaken.

Studies show that Imipenem is more efficient against* Acinetobacter *spp. [34] while Meropenem is more efficient against* Pseudomonas *spp. [35]. This fact explains why* Pseudomonas *spp. strains P4 and P5 were intermediately resistant to Meropenem while* Acinetobacter *spp. strains A3 and A5 were intermediately resistant to Imipenem. The difference among those 2 carbapenems fits the general trend of the MPC data obtained, with a few exceptions. Our data shows that strain A4 had exceptionally high MIC and MPC values with Imipenem (128 *μ*g/mL and 800 *μ*g/mL resp.) as compared to Meropenem (16 *μ*g/mL and 200 *μ*g/mL resp.).* Acinetobacter *spp. strains A2 and A5 also had higher MPC values with Imipenem (210 *μ*g/mL and 250 *μ*g/mL resp.) as compared to Meropenem (120 *μ*g/mL and 45 *μ*g/mL resp.). This may have been caused by the expression of certain types of MBL (such as IMP-1) that show a greater affinity to Imipenem rather than Meropenem in certain strains and not others [36]. The high MPC values detected in strains A4 and A5 may have also been caused by the expression of the MBL from an integron, with or without the added effect of changes in permeability [37]. Strains A2 and A3 both coexpressed MBL and OXA. However, strain A2 had higher MPC values than strain A3 for both antibiotics (Tables 4 and 5). Pertaining to the* Pseudomonas *spp. strains, the MPC values with Meropenem were lower than those with Imipenem. Both strains P4 and P5 were positive for KPC and MBL. However, the MPC values of strain P4 were higher than those for strain P5 (Tables 4 and 5). This difference could not be attributed to the mechanisms of resistance, per se, but to the subtype of the produced enzymes, the changes in permeability, the expression of MBL from an integron, and/or the amount of carbapenemases produced. Therefore, our data suggests that there is no direct correlation between a phenotypically detected mechanism of resistance and MPC values.

Further analysis of the MPC values revealed that the general trend is having higher MPC values for resistant strains as compared to susceptible ones. This data goes in line with the observation made by Drlica and Zhao which states that when resistance is acquired stepwise, the suppression of each successive mutant becomes increasingly more difficult [26]. Therefore, the MPC parameter may be of use at the beginning of the clinical treatment before allowing sufficient time for the bacteria to mutate as opposed to it being used to treat already resistant strains.

Tissue penetration studies have shown that the administration of 0.5 g to 1 g of Imipenem resulted in a concentration in excess of 4 *μ*g/mL in a wide variety of tissues that include the colonic, lung, pancreatic, peritoneal, bile, and ascetic tissues. 0.5 g to 1 g of Meropenem was also shown to be sufficient to deliver concentrations at the target tissue adequate enough to kill most bacteria [38]. These doses of antibiotics are able to deliver concentrations above the MIC at the target tissue. Nevertheless, the concentration obtained may still fall within the MSW zone, as is the case in the Meropenem sensitive* Pseudomonas *spp. strain P1. This strain shows MIC values with Meropenem of 2 *μ*g/mL. Strain P1 will be reported as Carbapenem sensitive and the physician may choose to treat the patient with Meropenem. Even though the administration of 0.5 g to 1 g of Meropenem will deliver concentrations of at least 4 *μ*g/mL at the target site, which is above the MIC, this concentration would fall in the MSW (since the MPC value with Meropenem for this strain is 14 *μ*g/mL). This would in turn encourage the emergence of resistant strains through the course of treatment and leads to an increased risk of treatment failure. However, in the case of strain P2, the administration of 0.5 g to 1 g of Meropenem would deliver concentrations above the MSW and therefore could be used without increasing the risk of developing bacterial resistance. On the other hand, achieving concentrations above the MSW for strains with high MPC values (such as strains A2, A4, A5, P4, and P5) is not possible since concentrations of carbapenems in excess of 4 grams per day are known to increase the risk of seizures [39]. In that case, the physician is encouraged to switch to another approach for treatment, such as combination therapy (such as Imipenem-colistin) [40] or to another antibiotic to which the microorganism remains susceptible. These examples are given in order to stress upon the observation that there is a unique MSW for every antibiotic-microorganism combination, as stated by Drlica and Zhao [26]. Therefore, if the MPC parameter is to be used, the unique MSW in combination with the specific antibiotic intended for use is to be determined.

Studies have shown that optimal bacterial killing for time-dependent antibiotics is when drug concentrations are maintained at 4 times the MIC through continuous infusion. The Time above the MIC (T>MIC) is what has been traditionally used in order to evaluate the efficacy of time-dependent antibiotics [41, 42]. The determined optimal conditions for bacterial killing could be due to the fact that this mode of administration is keeping the concentration of the antibiotic at values above the MSW and thus avoiding the emergence of resistance during therapy. One possible explanation for treatment failure, even if the guidelines were followed, could be because the drug concentrations at the target site are falling within the MSW and therefore selecting for resistant bacteria throughout the course of treatment. Since the determination of the MPC could be done in most clinical laboratories and the percentage of Imipenem and Meropenem binding to serum proteins is relatively low (<10% and 2%, resp.) [39, 42] (implying that the MPC data obtained in the laboratory could be of direct clinical use), we suggest adding the determination of the MPC to the panel of clinical laboratory tests for strains isolated from critically ill patients. The MSW could then be determined and the physician may choose in a more informed manner what course to take during treatment. If the MPC values fall within a safely administrable range, then it is also recommended to keep the antibiotic concentrations at values above the MPC throughout therapy and therefore use the Time above the Mutant Selection Window (T>MSW) instead of the T>MIC in order to evaluate the drug efficacy. However, if the MPC values prove to be too high for a particular isolate, then it is recommended to restrain from using carbapenems in order to avoid the risk of obtaining resistant strains during treatment.

In conclusion, no correlation between the phenotypic detection of the mechanisms of resistance and MPC values was observed in our study. The MPC values obtained were unique for every antibiotic-microorganism combination and therefore we recommend that testing for the MPC be implemented in the panel of laboratory tests when a critically ill person is to be given a course antibiotic treatment. If the MPC values fall within a safely administrable dose, the use of T>MSW instead of the T>MIC is also suggested in order to suppress the emergence of resistance during treatment. If the MPC values fall above safely administrable doses, then switching to another antibiotic is recommended, even if the MIC is achievable at the target site. The successful implementation of the MPC approach may not only suppress the emergence of bacterial resistance, but also safeguard these important antibiotics for future use.

## Figures and Tables

**Table 1:** Antibiotic Susceptibility Testing for *Acinetobacter baumannii*.

Antimicrobial agent	*Acinetobacter baumannii *									
	A1		A2		A3		A4		A5	
	*D* (mm)	S-I-R	*D* (mm)	S-I-R	*D* (mm)	S-I-R	*D* (mm)	S-I-R	*D* (mm)	S-I-R
Imipenem	19	S	6	R	9	R	6	R	9	R
Meropenem	19	S	6	R	8	R	9	R	8	R
Cefotaxime	6	R	6	R	6	R	21	I	6	R
Ceftazidime	6	R	6	R	6	R	28	S	6	R
Cefepime	6	R	6	R	6	R	28	S	6	R
Ertapenem	11	R	6	R	6	R	6	R	6	R
Gentamycin	7	R	6	R	6	R	31	S	9	R
Cefuroxime	6	R	6	R	6	R	6	R	6	R
Cefoxitin	6	R	12	R	12	R	6	R	15	R
Ciprofloxacin	6	R	6	R	6	R	29	S	9	R
Amoxicillin + clavulanic acid	6	R	6	R	6	R	7	R	6	R
Tigecycline	17	S	19	S	19	S	21	S	19	S
Tobramycin	16	S	11	R	10	R	25	S	10	R
Amikacin	16	I	13	R	10	R	32	S	11	R
Trimethoprim Sulfamethoxazole	6	R	6	R	6	R	21	S	6	R
Ampicillin	6	R	6	R	6	R	6	R	6	R

“S” stands for susceptible, “I” for intermediate resistance, and “R” for resistant. “*D*” stands for diameter of inhibition zone. A1 through A5 represent the 5 *Acinetobacter baumannii* strains studied. The test was done according to CLSI guidelines.

**Table 2:** Antibiotic Susceptibility Testing for *Pseudomonas *spp.

Antimicrobial agent	*Pseudomonas *spp.									
	P1		P2		P3		P4		P5	
	*D* (mm)	S-I-R	*D* (mm)	S-I-R	*D* (mm)	S-I-R	*D* (mm)	S-I-R	*D* (mm)	S-I-R
Imipenem	22	S	23	S	25	S	9	R	11	R
Meropenem	14	S	18	S	31	S	14	R	8	R
Aztreonam	21	I	14	R	26	S	22	S	19	I
Ceftazidime	22	S	14	R	26	S	20	S	13	R
Gentamycin	19	S	6	R	24	S	15	S	15	S
Ciprofloxacin	30	S	6	R	25	S	24	S	14	R
Piperacillin/Tazobactam	25	S	20	S	26	S	21	S	13	R
Piperacillin	23	S	17	R	11	R	21	S	12	R
Tobramycin	21	S	7	R	24	S	17	S	19	S
Amikacin	23	S	17	S	22	S	17	S	17	S
Trimethoprim Sulfamethoxazole	6	R	6	R	12	R	6	R	6	R

“S” stands for susceptible, “I” for intermediate resistance, and “R” for resistant. “*D*” stands for diameter of inhibition zone. P1 through P5 represent the 5 *Pseudomonas *spp. strains studied. The test was done according to CLSI guidelines.

**Table 3:** Results of the phenotypic tests.

Phenotypic tests							
Strain	ESBL	Chromosomal AmpC	Plasmidic AmpC	MBL	KPC	OXA	CCCP
A1	−	−	−	−	−	−	−
A2	−	−	−	+	−	+	−
A3	−	−	−	+	−	+	−
A4	+	−	−	+	−	−	−
A5	−	−	−	+	−	−	−
P1	−	−	−	−	−	−	−
P2	−	−	−	−	−	−	−
P3	−	+	−	−	−	−	−
P4	−	+	−	+	+	−	−
P5	−	+	+	+	+	−	−

“ESBL” stands for extended spectrum *β*-lactamase, “MBL” stands for Metallo-*β*-lactamase, “KPC” stands for Klebsiella Pneumoniae Carbapenemase, “OXA” stands for Oxacillinase, and “CCCP” stands for Carbonyl Cyanide m-Chlorophenylhydrazone.

**Table 4:** Determination of MIC, MPC, and MSW using Imipenem.

Isolate no.	MIC		Average MIC	MPC	Average MPC	MSW	Average MSW
	μg/mL	S-I-R	(μg/mL)	(μg/mL)	(μg/mL)	(μg/mL)	(μg/mL)
*Acinetobacter *							
A1	2	S	35.6 ± 52.92	4	255.2 ± 324.47	2	219.6 ± 274.19
A2	32	R		210		178	
A3	8	I		12		4	
A4	128	R		800		672	
A5	8	I		250		242	
*Pseudomonas *							
P1	4	S	7.5 ± 7.87	30	48 ± 45.14	26	40.5 ± 37.56
P2	1	S		16		15	
P3	0.5	S		4		3.5	
P4	16	R		110		94	
P5	16	R		80		64	

S-I-R stands for susceptible, intermediate, and resistant, respectively.

**Table 5:** Determination of MIC, MPC, and MSW using Meropenem.

Isolate no.	MIC		Average MIC	MPC	Average MPC	MSW	Average MSW
	μg/mL	S-I-R	(μg/mL)	(μg/mL)	(μg/mL)	(μg/mL)	(μg/mL)
*Acinetobacter *							
A1	4	S	23.2 ± 12.77	70	101 ± 61.68	66	77.8 ± 65.77
A2	32	R		120		88	
A3	32	R		70		38	
A4	16	R		200		184	
A5	32	R		45		13	
*Pseudomonas *							
P1	2	S	3.8 ± 3.88	14	13.5 ± 11.48	12	9.7 ± 8.23
P2	0.5	S		1.5		1	
P3	0.5	S		4		3.5	
P4	8	I		30		22	
P5	8	I		18		10	

S-I-R stands for susceptible, intermediate, and resistant, respectively.
